# Coronal plane alignment of the knee (CPAK) classification and its impact on medial unicompartmental knee arthroplasty: exposing a unexpected external shift of limb mechanical axis in case of prearthritic constitutional valgus alignment: a retrospective radiographic study

**DOI:** 10.1186/s43019-024-00217-6

**Published:** 2024-03-30

**Authors:** Vitantonio Digennaro, Riccardo Ferri, Alessandro Panciera, Barbara Bordini, Davide Cecchin, Lorenzo Benvenuti, Francesco Traina, Cesare Faldini

**Affiliations:** 1 Clinica Ortopedica e Traumatologica I, IRCCS Istituto Ortopedico Rizzoli, Via Giulio Cesare Pupilli 1, 40136 Bologna, Italy; 2 Laboratorio di Tecnologia Medica, IRCCS Istituto Ortopedico Rizzoli, Via Giulio Cesare Pupilli, 1, 40136 Bologna, Italy; 3 Ortopedia-Traumatologia e Chirurgia Protesica e Dei Reimpianti D’anca e di Ginocchio, IRCCS Istituto Ortopedico Rizzoli, Via Pupilli 1, 40136 Bologna, Italy

**Keywords:** Unicompartmental, Knee, aHKA, CPAK, LDFA, Alignment

## Introduction

Unicompartmental knee arthroplasty (UKA) accounts for 8–12% of all knee arthroplasties, with 90% of cases involving the medial compartment [1]. Clinical and functional outcomes of medial UKA have progressively improved over time, due to better and reproducible surgical techniques, refinements in prosthetic design, surgeon’s experience and patient selection, with survival rates of 85–98% at 10 years after surgery widely reported in literature [2–5].

Since Marmor introduced it in 1972 [6, 7], there has been a significant advancement of the clinical indications in medial UKA. Specifically, with Kozinn and Scott initial contribution in 1989 [8] and finally reaching a large consensus with most authors these days. On the other hand, the radiographic indications in medial UKA have remained nearly the same in the last decades, based on an overall assessment of the limb’s alignment and evaluating the tibial deformity mainly with the Cartier angle [9–11].

Currently, no previous studies have considered deformity of the distal femur and prearthritic constitutional alignment as selection criteria that allow to perform a medial UKA.

The coronal plane alignment of the knee (CPAK) classification [12] has highlighted that similar knee deformities could be explained by several underlying femoral and tibial features: nine types of constitutional alignment of the knee were delineated, by introducing the independent variables of arithmetic hip–knee–ankle angle (aHKA, with varus, neutral, and valgus subgroups) and joint line obliquity (JLO, with apex distal, neutral, and apex proximal subgroups) [12–14].

According to the CPAK [12], the lower limb mechanical axis shifts throughout the Osteoarthritis (OA) development, usually accentuating the prearthritic constitutional alignment, but occasionally this can be inverted; thus, a constitutional valgus alignment potentially can shift to varus due to the medial joint space loss [12].

## Objectives

This retrospective radiographic study focuses on knees with prearthritic constitutional valgus alignment, primarily related to a valgus epiphyseal deformity of the distal femur and delineated by positive values of aHKA, with JLO respectively apex distal (type III), neutral (type VI), and apex proximal (type IX) [12–14]. The first purpose was to assess the amount of prearthritic constitutional valgus alignment among patients with isolated medial OA undergoing medial UKA. Moreover, we investigated whether this particular condition could have an impact on post-operative alignment. In fact, we suspected that an undesirable external shift of limb’s mechanical axis could occur in case of constitutional valgus aligment, due to the restoration of the medial compartment height with medial UKA and the limb’s return to its prearthritic alingnment. This could lead to OA progression in the lateral unaffected compartment as suggested by the recent literature[4, 15, 16]. Specifically, we examined the displacement of limb’s mechanical axis from pre- to postoperative, both in its coronal relationship with the center of the knee, and in the magnitude of variation in mechanical HKA (mHKA) and mechanical axis deviation (MAD).

## Materials and methods

### Participants

A total of 188 consecutive patients treated by medial UKA between January 2018 and December 2022 in two operating units of our department were retrospective evaluated. Nine patients were excluded due to the absence of adequate radiographs, resulting in a final inclusion of 179 patients, 85 left knees (47.5%), and 94 right knees (52.5%).

The average age of included patients at the time of surgery was 68.9 ± 2.46 years (range 53–86 years), 84 men (47.2%), and 95 women (52.8%). The mean BMI was 27.9 ± 1.38 kg/m^2^ (range 20.3–39.427.9 ± 1.38 kg/m^2^).

All patients presented appropriate clinical indications to medial UKA, including varus deformity less than 15° easily correctable by passive valgus-stress [17, 18], elective pain confined to the medial compartment without lateral or patellofemoral symptoms [2, 6, 7], preserved articular stability [19], and absence of persistent extension impairment [18]. Of the patients, 174 (97.3%) showed isolated medial OA (Ahlbäck stage III–IV) [20], while in five cases (2.7%) an osteonecrosis of medial femoral condyle or tibial plateau was the main diagnosis [21]. Radiographic criteria for tibial deformity allowing for medial UKA were respected in all patients, primarily described by a Cartier angle lower than 5° [2, 11]. The presence of mild patellar osteophytes did not represent a contraindication to the procedure in absence of related symptomatology [22–24]. Patients of advanced age [25, 26], with elevated BMI [27, 28] and chondrocalcinosis [29, 30] were not excluded as well, according to recent literature recommendations.

Of the patients, 55 (30.5%) underwent previous partial or total medial meniscectomy, while no previous high tibial osteotomy (HTO) or anterior cruciate ligament (ACL) reconstruction were carried out before the arthroplasty procedure.

### Radiographic evaluation and investigated data

Weight-bearing long leg radiographs were performed preoperatively, after surgery during the first recovery week, and subsequently at 1, 3, 6, and 12 months of follow-up. Additionally, weight bearing laterolateral, Rosemberg view, and patella axial radiographs were obtained preoperatively to ensure the correct indication to medial UKA.

All-evaluations were obtained by well-trained observers via the electronic picture archiving and communication system (Philips CARESTREAM Vue PACS, accuracy 0,1°) used at our clinic. The intraobserver reliability and inter-observer reliability were assessed using the interclass correlation coefficient (ICC) [31]. Initially, all evaluations were performed by one author and then were remeasured more than 1 week later by the same observer and also by two other observers. The intraobserver correlation between the first and second evaluation performed by the first author was 0.87. The interobserver correlation between the second evaluation acquired by different authors was 0.82. None of the observers partecipated in the surgical operations.

On preoperative long leg radiographs, the following parameters were evaluated: mechanical hip–knee–ankle angle (mHKA), defined as the angle between femoral and tibial mechanical axes (MA), with negative values associated to varus and positive values to valgus [32]; mechanical axis deviation (MAD), defined as the perpendicular distance between the MA of the lower extremity and the center of the knee joint, with negative values associated to varus and positive values to valgus [32]; lateral distal femur angle (LDFA), defined as the angle open laterally formed by the MA of the femur and the line tangent to the distal surface of femoral condyles, with values > 90° associated to varus and < 87,5° to valgus [32]; medial proximal tibial angle (MPTA), defined as the angle open medially formed by the MA of the tibia and the line tangent to the proximal surface of tibial plate, with values < 87,5° associated to varus and > 90° to valgus [32]; joint line congruence angle (JLCA), defined as the angle between the lines tangent to the articular surfaces of distal femur and proximal tibia [32]; Cartier angle, defined as the angle formed by the perpendicular to the line tangent to the lateral tibial plateau and MA of the tibia [9–11]. Additionally, we highlighted for all patients the prearthritic constitutional alignment of the lower limbs using arithmetic HKA (aHKA) and joint line obliquity (JLO), as suggested by the CPAK classification [12–14]. The aHKA was calculated by subtracting the LDFA from MPTA, with negative, zero, and positive values associated respectively to constitutional varus, neutral, and valgus alignment. The JLO was instead calculated by adding the MPTA to LDFA, with values < 180°, 180°, and > 180°, associated respectively to apex distal, neutral, and apex proximal obliquity. As described by the authors, these evaluations are highly accurate and reproducible in patients with deformities less than or equal to 8° and absence of bone loss at the contact points, as is typical in patients with indication to medial UKA [12–14].

On postoperative long leg radiographs, the following parameters were evaluated: coronal femoral component angle (c-FCA), defined as the angle between the line tangent to the femoral component distal cut and MA of the femur, with negative values associated to varus and positive values to valgus [15]; coronal tibial component angle (c-TCA), defined as the angle between the line tangent to the tibial component coronal cut and MA of the tibia, with negative values associated to varus and positive values to valgus [15]; mechanical hip-knee-ankle angle (mHKA), defined as above; mechanical axis deviation (MAD), defined as above; ΔHKA, defined as the difference between preoperative mHKA and postoperative mHKA expressed as absolute value; ΔMAD, defined as the difference between preoperative MAD and postoperative MAD expressed as absolute value.

The first goal was to assess the amount of constitutional valgus alignment among the included patients affected by isolated medial OA treated by medial UKA.

Subsequently, the displacement of limb’s mechanical axis from pre- to postoperative was assessed, both in terms of its coronal relationship with the center of the knee and the magnitude of variation in mHKA and MAD resulting from the procedure. These data were analyzed by comparing patients with constitutional varus alignment (negative values of aHKA) and those with constitutional valgus alignment (positive values of aHKA).

### Surgical procedures

All surgeries were performed by two experienced surgeons using two prosthetic designs of medial UKA from Smith + Nephew (Watford, Hertfordshire, United Kingdom), both metal-backed: 105 Journey Uni (58.9%) and 74 Journey II UK (41.1%). The advantage of using prostheses of the same manufacturer is the overlapping component design, which allows us to carry out comparable measurements of radiographic prosthetic positioning and limb alignment.

Medial UKA procedures were performed using a minimally invasive mid-vastus approach, according to the Cartier technique [2, 11]: to achieve a kinematic alignment with an anatomical tibial cut referenced to the native metaphyseal axis and the femoral component therefore aligned cylindrically with reference to the tibial cut [2, 33, 34]. This alignment technique is also recommended by manufacturers to optimize contact points between the prosthetic components throughout the range of motion [35]. UKA procedures involved intraarticular augmentation to compensate for cartilage wear, without any correction of the bone deformity and using the minimum polyethylene size necessary to achieve optimal ligament balancing while avoiding overcorrection [33, 36]. Thus, the polyethylene size has been reported for all procedures, as it is a contributing factor in the medial compartment height after the procedure.

### Statistical analysis

The study variables were analyzed and compared among groups using nonparametric Mood’s median tests (SPSS 14.0, version 14.0.1; SPSS Inc, Chicago, IL). Significance was set at *P* value < 0.05.

Ethical approval for the study has not been necessary since no additional clinical procedures have been performed beside the standard radiographic evaluation, and the anonymity of patients was preserved.

All patients provided written informed consent for the inclusion in the study, in accordance with the Ethical Standards of the 1964 Declaration of Helsinki, as revised in 2000.

## Results

### Preoperative radiographic evaluation

Table 1 shows mean value and range of main preoperative radiographic evaluation of the included patients, measured on long leg radiographs.

**Table 1:** Preoperative radiographic evaluation

LDFA	88.5° ± 0.42° (range 84.3–93.9°)
MPTA	86.8° ± 0.43° (range 83.5–90.6°)
JLCA	4.27° ± 0.32° (range 0.89–8.51°)
Cartier angle	2.56° ± 0.37° (range 0.15–5.02°)
aHKA	−2.68° ± 0.62° (range −7.02° to +5.4°)
JLO	175.7° ± 0.54° (range 170.8–182.3°)
mHKA	−6.12° ± 0.71° (range −12.93° to −0.16°)
MAD	−2.28 ± 0.32 cm (range −6.01 to −0.42 cm)

### Amount of constitutional valgus alignment

Among the 179 included patients with isolated medial OA treated by medial UKA, 16 of them (8.92%) presented a constitutional valgus alignment, due to valgus values of LDFA (range 84.2–86.4°) and positive values of aHKA (range +0.5° to +5.8°). All these 16 patients presented with an apex distal JLO (range 171.7–174.2°), thus belonging to type III subgroup of the CPAK classification. None of these patients with constitutional valgus alignment belonged to type VI (neutral JLO) or IX (apex proximal JLO).

These cases consisted of 4 men (24.3%) and 12 women (75.7%), in line with the previously established higher rate of constitutional valgus alignment among women [13]. Furthermore, 6 of these patients (37.9%) had previously undergone medial meniscectomy at an earlier time.

### Alignment of prosthetic components and polyethylene size

The alignment of prosthetic components measured on postoperative long-leg radiographs yielded the following results: c-TCA −2.97 ± 0.81 (range −0.12 to −5.99) and c-FCA −1.29 ± 0.89 (range −6.88 to +4.51).

The polyethylene size used most frequently was 8 mm in 129 patients (72.2%), followed by 9 mm in 34 patients (18.9%), 10 mm in 10 patients (5.34%), and 11 mm in 6 patients (3.56%).

### Displacement of limb’s mechanical axis

The displacement of limb’s mechanical axis from pre- to postoperative was assessed both in terms of its coronal relationship with the center of the knee and the absolute value of modification in mHKA and MAD resulting from the medial UKA.

**Fig. 1:**
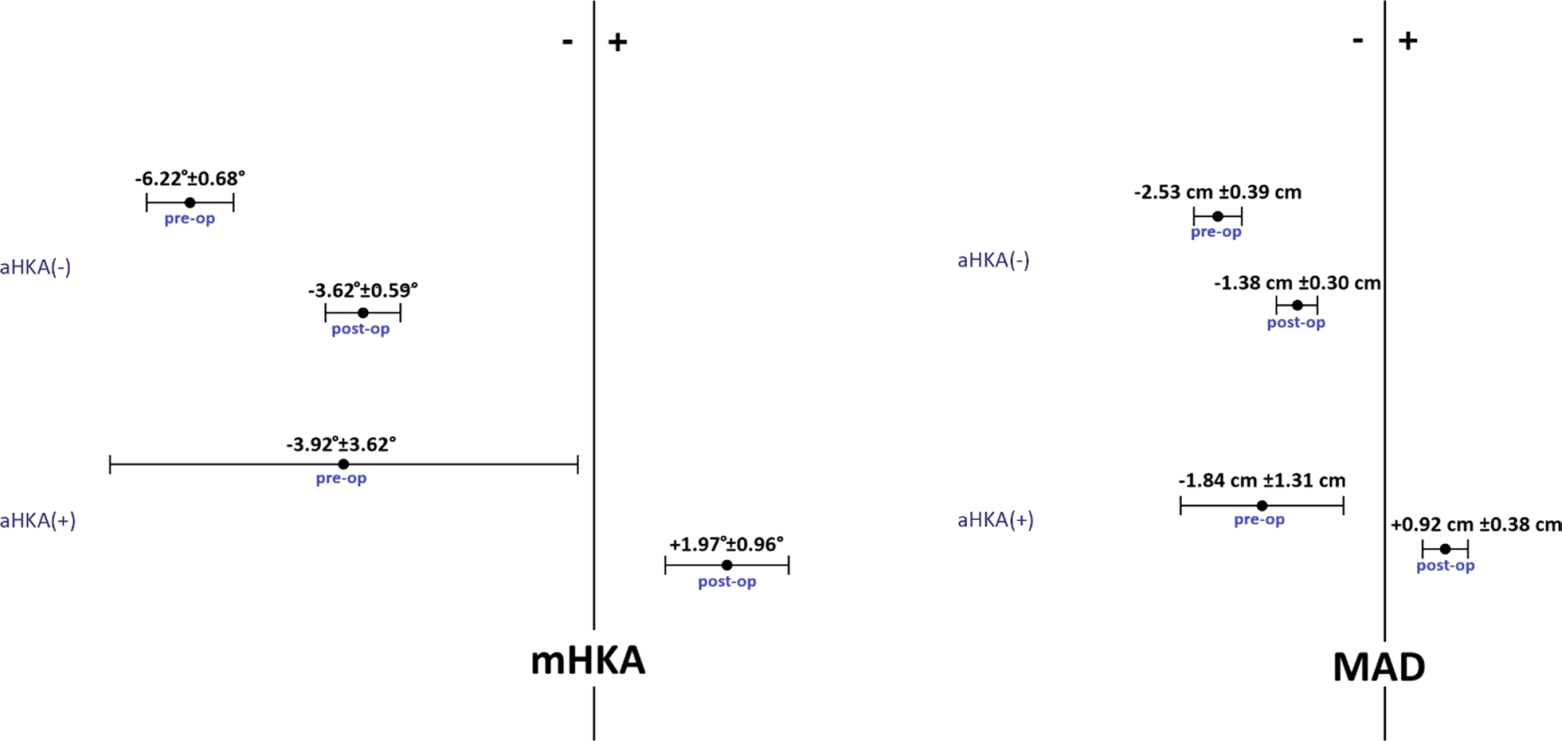
Displacement of limb’s mechanical axis from pre- to postoperative, showing the mean value and range of mHKA and MAD, subdivided in patients with constitutional varus alignment (negative values of aHKA) and constitutional valgus alignment (positive values of aHKA)

**Fig. 2:**
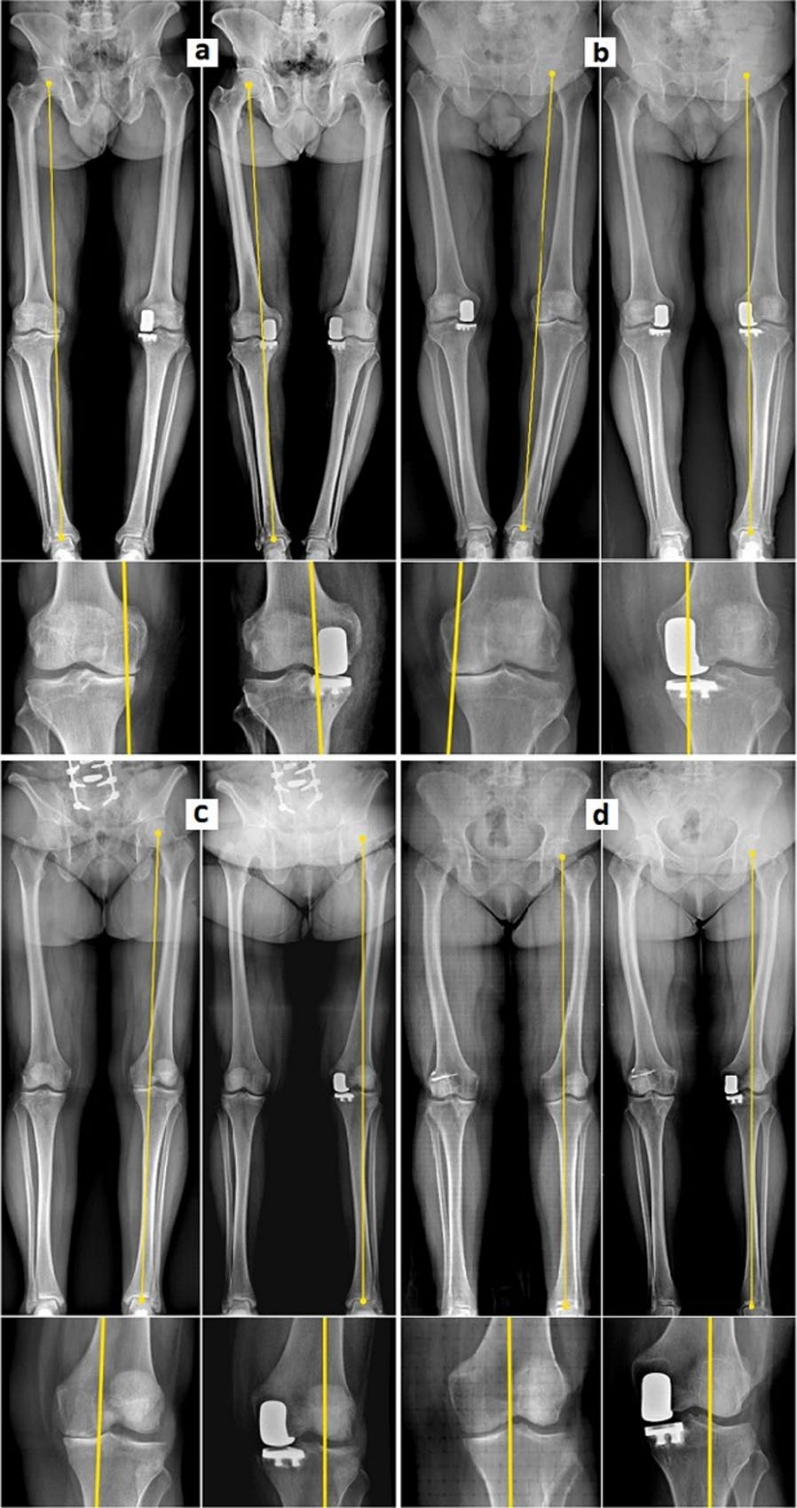
Displacement of limb’s mechanical axis in long-leg standing radiographs from pre- to postoperative, subdivided in patients with constitutional varus alignment (negative values of aHKA) **a**, **b** and constitutional valgus alignment (positive values of aHKA) (**c**, **d**). It can be observed that in presence of constitutional varus alignment **a**, **b** the limb’s mechanical axis remains internal to the center of the knee despite the recovery of medial compartment height. Conversely, in presence of constitutional valgus alignment (**c**, **d**) the limb’s mechanical axis shifts from internal to external, restoring the valgus alignment that was present prior to the OA onset

The key finding of our study was the discovery that all 16 patients with constitutional valgus alignment (positive values of aHKA) experienced a shift of the limb’s mechanical axis from internal relative to the center of the knee preoperatively to external postoperatively. This shift occurred due to the restoration of the medial compartment height without any overcorrection, thereby returning the limb to its constitutional valgus alignment that was present prior to the OA onset. On the other hand, in all 163 patients with constitutional varus alignment (negative values of aHKA), the limb’s mechanical axis consistently remained internal relative to the center of the knee from pre- to postoperative. (Table 2; Figs. 1,2).

**Table 2:** Mean value and range of pre- and postoperative mHKA and MAD

	Preoperative mHKA	Postoperative mHKA	Preoperative MAD	Postoperative MAD
Overall patients	−6.12° ± 0.71° (−12.93° to −0.16°)	−3.18° ± 0.61°(−9.82° to +3.82°)	−2.28 cm ± 0.32 cm (−6.01 to −0.42 cm)	−1.12 cm ± 0.34 cm (−3.65 to +1.41 cm)
Varus aHKA (−)	−6.22° ± 0.68° (−12.93° to −1.13°)	−3.62° ± 0.59°(−9.82° to −0.25°)	−2.53 cm ± 0.39 cm (−6.01 to −0.42 cm)	−1.38 cm ± 0.30 cm (−3.65 to −0.18 cm)
Valgus aHKA ( +)	−3.92° ± 3.62° (−9.18° to −0.16°)	+1.97° ± 0.96°(+1.14° to +3.82°)	−1.84 cm ± 1.31 cm (−3.31 to −0.53 cm)	+ 0.92 cm ± 0.38 cm (+0.52 cm to +1.41 cm)

In addition, we have demonstrated that in all 16 patients with constitutional valgus alignment, the magnitude of axial displacement, in terms of absolute value of ΔHKA and ΔMAD, was greater compared with those with constitutional varus alignment (mean ∆HKA and ∆MAD were 5.79° ± 3.10° vs. 3.17° ± 0.51° and 2.81 cm ± 1.12 cm vs. 1.34 cm ± 0.28 cm, respectively) (Table 3).

**Table 3:** Displacement of limb’s mechanical axis in terms of absolute value of ΔHKA and ΔMAD

	ΔHKA	ΔMAD
Overall patients	3.36° ± 0.51° (0.13°/9.72°)	1.51 cm ± 0.27 cm (0.06/4.21 cm)
Varus aHKA (-)	3.17° ± 0.51° (0.13°/8.99°)	1.34 cm ± 0.28 cm (0.06/3.68 cm)
Valgus aHKA ( +)	5.79° ± 3.10° (1.71°/9.72°)	2.81 cm ± 1.12 cm (1.65/4.21 cm)

**Fig. 3:**
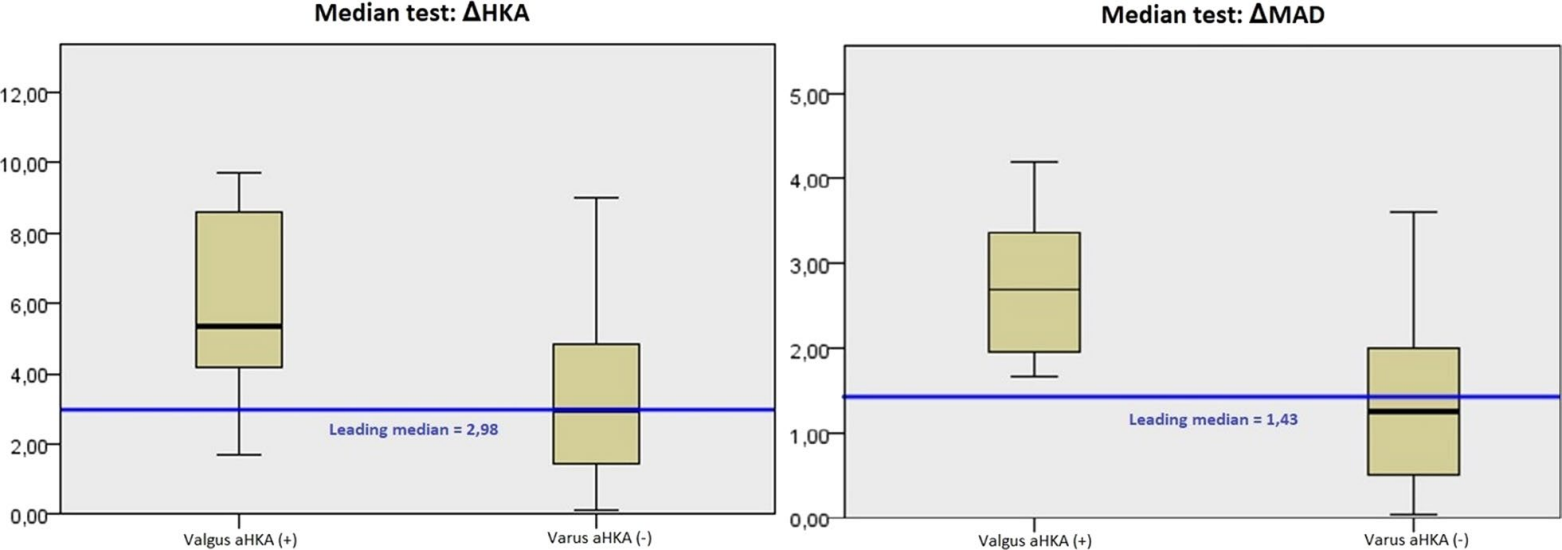
Amount of axial displacement in patients with constitutional valgus alignment compared with those with constitutional varus alignment, assessed using nonparametric Mood’s median tests

The average amount of axial displacement in patients with constitutional valgus alignment was generally greater compared with those with constitutional varus alignment, as observed in terms of absolute value of ΔHKA (*P* = 0.203) and with statistically significant differences in ΔMAD (*P* = 0.024) (Fig. 3).

## Discussion

The main finding of the present study was that among the 179 patients with isolated medial OA undergoing medial UKA, 16 of them (8.92%) presented a prearthritic constitutional valgus alignment. This alignment was entirely described by valgus values of LDFA (range 84.2–86.4°) and positive values of aHKA (range +0.5° to +5.8°). All 16 patients presented with an apex distal JLO (range 171.7–174.2°).

Nowadays, the radiological criteria for medial UKA indication are represented by the overall assessment of limb deviation and tibial deformity using the Cartier angle [9–11]. Thus, in our study, we have expanded the preoperative radiographic planning to include the evaluation of the distal femur and the prearthritic constitutional alignment.

According to the CPAK classification [12], constitutional valgus alignment is primarily associated with a valgus epiphyseal deformity of distal femur and represents a minority among the different alignment types of the knee. It includes the type III (positive aHKA, apex distal JLO), type VI (positive aHKA, neutral JLO), and type IX (positive aHKA, apex proximal JLO), respectively, accounting for 9.8%, 3.4%, and 0.2% of the overall healthy population.

With the progression of OA, the narrowing of medial joint space can significantly alter the limb mechanical alignment over time: usually, the alignment present at skeletal maturity is emphasized. Occasionally, there can be an inversion where a constitutional valgus alignment potentially shifts to varus [12, 13]. In our series, previous surgical interventions may have represented a determining factor in this alignment inversion. Specifically, 6 of 16 patients (37.9%) with medial OA and constitutional valgus alignment underwent previous partial or total medial meniscectomy at an earlier time.

Our study demonstrated that in all 16 patients with isolated medial OA and constitutional valgus alignment, the limb’s mechanical axis shifted from internal to external in relation to the center of the knee following medial UKA. This shift occurred during the restoration of the medial compartment height without any overcorrection, thereby restoring the limb’s constitutional valgus alignment that was present before the development of OA.

Currently, there is a widespread consensus among authors that the overcorrection of coronal deformity in medial UKA is a major risk factor for a rapid progression of OA in the lateral unaffected compartment [4, 15, 16]. Therefore, the appropriate indication for medial UKA in patients with constitutional valgus alignment becomes a critical question.

Indeed, literature has demonstrated that an external axial shift associated with 5° of overcorrection transfers 88% of the loads to the lateral compartment; this could lead to rapid development of external symptoms and clinical failure, requiring an inevitable revision arthroplasty, often within 2 years of the surgery [3, 10, 36, 37].

Additionally, in all 16 patients with constitutional valgus alignment, the magnitude of axial displacement in terms of ΔHKA and ΔMAD has been greater than those with constitutional varus alignment (mean ∆HKA and ∆MAD, respectively, 5.79° ± 3.10° versus 3.17° ± 0.51° and 2.81 cm ± 1.12 cm versus 1.34 cm ± 0.28 cm). This difference is likely connected to complex morphological factors besides the coronal plane alignment.

In fact, according to the CPAK classification [12], constitutional valgus knees often exhibit intrinsic alterations in the lateral epiphyses of femur and tibia, with bone deficiencies and external rotation, as well as periarticular soft tissue alterations, including attenuation of medial ligament structures and contractures of the lateral ones [12, 13]. This subsequent external standing imbalance leads to a more complex reconstructive solution that goes beyond the restoration of constitutional alignment, particularly in unicompartmental procedures, where a kinematic alignment should be performed while respecting the ligament balance of the knee, without possibility of modification [11, 38].

Therefore, based on the results of our study, patients with isolated medial OA undergoing medial UKA could have an increased risk of progression of OA in the lateral unaffected compartment and may fail to achieve an appropriate ligament balance in case of constitutional valgus alignment.

However, all studies of external overloading following medial UKA have been performed in patients with medial OA and a prearthritic varus alignment [4, 15, 16]. It is therefore necessary to determine whether the recovery of compartment height with the external shift of the mechanical axis should be considered as an overcorrection in presence of prearthritic valgus alignment. In these patients, the valgus alignment represents the constitutional alignment, and its restoration might not necessarily alter the load distribution between compartments.

A limitation of this study lies in the small number of patients with isolated medial OA presenting constitutional valgus alignment compared with their counterparts with constitutional varus alignment.

This disparity is in line with the classification provided by the CPAK [12], which highlights that constitutional valgus alignment represents a minority among the different spectrum of knee alignment patterns. Additionally, the occurrence of patients with constitutional valgus alignment who subsequently develop isolated medial OA necessitating medial UKA is even rarer.

In our study, the external shift of the mechanical axis following medial UKA in patients with constitutional valgus alignment was demonstrated with statistical significance, highlighting the strength of the hypothesis despite the numerical disparity between the two groups.

While our radiographic results are indeed significant and we have introduced intriguing insights with possible clinical correlations as suggested in recent literature, further clinical studies are necessary to substantiate clinical correlations that stem from these radiographic observations.

Our radiographic examination was able to yield statistically significant results despite the limited number of patients with constitutional valgus alignment, since its foundation on reproducible and objective radiographic parameters. However, subsequent studies aiming at establishing clinical correlations on this topic will require a larger sample size to ensure statistical significance. It would be advisable to conduct studies with various follow-up periods and preferably involving multiple medical centers to enhance the likelihood of encompassing a larger pool of cases among these rare instances of patients, challenging to collect in sufficient numbers within a single center, even among hospitals with high volumes.

## Conclusions

Considering the current comprehensive knowledge about knee deformities and the individual variability of the OA development, it appears necessary to expand the radiological criteria for medial UKA, beyond the traditional assessment of limb deviation and tibial deformity.

According to our study, the evaluation of epiphyseal deformity of the distal femur and constitutional alignment could help to identify patients who may not achieve an appropriate postoperative alignment and ligament balance following medial UKA, despite having isolated medial OA and meeting all other indication criteria.

## Data Availability

the data may be provided upon request to the corresponding author.
